# Transient Expression in *Nicotiana Benthamiana* Leaves for Triterpene Production at a Preparative Scale

**DOI:** 10.3791/58169

**Published:** 2018-08-16

**Authors:** Michael J. Stephenson, James Reed, Bastiaan Brouwer, Anne Osbourn

**Affiliations:** ^1^John Innes Centre

**Keywords:** Biochemistry, Issue 138, *Nicotiana benthamiana*, vacuum infiltration, transient expression, agroinfiltration, triterpenes, hyper-translatable Cow Pea Mosaic Virus protein expression system

## Abstract

The triterpenes are one of the largest and most structurally diverse families of plant natural products. Many triterpene derivatives have been shown to possess medicinally relevant biological activity. However, thus far this potential has not translated into a plethora of triterpene-derived drugs in the clinic. This is arguably (at least partially) a consequence of limited practical synthetic access to this class of compound, a problem that can stifle the exploration of structure-activity relationships and development of lead candidates by traditional medicinal chemistry workflows. Despite their immense diversity, triterpenes are all derived from a single linear precursor, 2,3-oxidosqualene. Transient heterologous expression of biosynthetic enzymes in *N. benthamiana* can divert endogenous supplies of 2,3-oxidosqualene towards the production of new high-value triterpene products that are not naturally produced by this host. Agro-infiltration is an efficient and simple means of achieving transient expression in *N. benthamiana*. The process involves infiltration of plant leaves with a suspension of *Agrobacterium tumefaciens *carrying the expression construct(s) of interest. Co-infiltration of an additional *A. tumefaciens* strain carrying an expression construct encoding an enzyme that boosts precursor supply significantly increases yields. After a period of five days, the infiltrated leaf material can be harvested and processed to extract and isolate the resulting triterpene product(s). This is a process that is linearly and reliably scalable, simply by increasing the number of plants used in the experiment. Herein is described a protocol for rapid preparative-scale production of triterpenes utilizing this plant-based platform. The protocol utilizes an easily replicable vacuum infiltration apparatus, which allows the simultaneous infiltration of up to four plants, enabling batch-wise infiltration of hundreds of plants in a short period of time.

**Figure:**
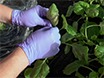


## Introduction

The triterpenes are one of the largest and most structurally diverse families of plant natural products. Despite their immense diversity, all triterpene natural products are believed to be derived from the same linear precursor 2,3-oxidosqualene, a product of the mevalonate pathway in plants. Cyclization of 2,3-oxidosqualene is initiated and controlled by a family of enzymes termed oxidosqualene cyclases (OSCs). This cyclization step represents the first level of diversification, with hundreds of different triterpene scaffolds having been reported from nature^1^. These scaffolds are further diversified by tailoring enzymes including, but not limited to, cytochrome p450s (CYP450s)^1^,^2^. Such biosynthetic pathways can lead to immense complexity, sometimes resulting in final products which are barely recognizable from the parent triterpene. For example, the complex structure of the potent insecticidal and antifeedant limonoid azadirachtin is believed to derive from a tetracyclic triterpene of the tirucallane type^3^.

Many triterpene-derived natural products, even those with relatively unmodified parent scaffolds, have been shown to possess medicinally relevant biological activity^4^,^5^,^6^,^7^. However, thus far this potential has not translated into a plethora of triterpene-derived drugs in the clinic. This is arguably (at least partially) a consequence of limited practical synthetic access to this class of compound, a problem that can stifle the exploration of structure-activity relationships and development of lead candidates by traditional medicinal chemistry workflows.

Transient expression of triterpene biosynthetic enzymes from other plant species in *N. benthamiana* leaves can divert endogenous supplies of 2,3-oxidosqualene towards the production of new high-value triterpene products (Figure 1). This process can be used to functionally characterize candidate enzymes and reconstruct the biosynthetic pathways of important naturally-occurring metabolites. Equally, it can also be exploited in combinatorial biosynthetic approaches to produce novel triterpene products, a strategy that can result in libraries of structurally related analogs, allowing systematic exploration of the structure-activity relationships of biologically active lead compounds^8^,^9^.

Agroinfiltration is an efficient and simple means of achieving transient expression in *N. benthamiana* leaves. The process involves the infiltration of leaves with a suspension of *A. tumefaciens* carrying the binary expression construct(s) of interest. This is achieved via the application of pressure which forces the liquid through the stomata, displacing air in the intercellular space, and replacing it with the *A. tumefaciens* suspension. The bacteria transfer the respective T-DNAs to the interior of the plant cells, resulting in localized and transient protein expression in the infiltrated leaf tissue.

While any binary vector suitable for generating transgenic plants may be employed for transient expression, we utilize the Cowpea Mosaic Virus (CPMV)-derived *Hypertranslational* (*HT*) protein expression system^10^,^11^. In this system the gene of interest is flanked by untranslated regions (UTR) from the CPMV RNA-2. The 5' UTR contains modifications resulting in very high levels of protein translation with no reliance on viral replication^12^. This technique has been developed into the Easy-And-Quick (pEAQ) binary vector series which includes site-specific recombination cloning protocol-compatible constructs (pEAQ-*HT*-DEST)^10^,^11^. Most pEAQ vectors also contain a tomato bushy stunt virus-derived P19 silencing suppressor gene^13^ within the T-DNA portion of the expression cassette, which circumvents the need to coinfiltrate a separate P19-carrying strain and affords very high-level protein expression in the host plant cell^10^,^11^.

Use of *N. benthamiana* as an expression host has particular advantages when working with plant biosynthetic pathways. The cell architecture intrinsically supports appropriate mRNA and protein processing, and proper compartmentalization, in addition to possessing the necessary co-enzymes, reductases (for CYP450s), and metabolic precursors. The carbon source is photosynthesis; thus, plants can simply be grown in good quality compost, requiring only water, CO_2_ (from the air), and sunlight as inputs. The platform is also extremely convenient for co-expression of different combinations of proteins, as this can be achieved facilely by the co-infiltration of different strains of *A. tumefaciens,* negating the need to build large multigene expression cassettes. Furthermore, the process can be linearly and reliably scaled simply by increasing the number of plants used in the experiment.

Previous work in our laboratory has demonstrated the utility of this platform for preparative scale experiments. This included the preparation of novel triterpenes for use in bioactivity assays and scale up to achieve gram quantities of isolated product. Furthermore, accumulation of heterogenous triterpene products can be increased several fold by co-expression of an N-terminal-truncated, feedback-insensitive form of 3-hydroxy, 3-methyglutary-coenzyme A reductase (tHMGR), a rate-limiting upstream enzyme in the mevalonate pathway^8^.

Key to such preparative-scale experiments is the ability to conveniently up-scale the infiltration process. In a typical experiment, tens to hundreds of plants may be required to achieve the target quantity of isolated product. Infiltrating individual leaves by hand (using a needless syringe) is operationally demanding, and often prohibitively time-consuming, rendering this method impractical for routine scale-up. Vacuum infiltration offers advantages over hand infiltration, as it is not dependent on the skill level of the operator and allows the infiltration of a greater area of the leaf surface. This procedure is used commercially for the large-scale production of pharmaceutical proteins^14^. This protocol utilizes an easily replicable vacuum infiltration apparatus, which can be constructed from commercially available parts. This allows the simultaneous infiltration of up to 4 plants, affording rapid and practical batch-wise infiltration of hundreds of plants in a short period of time (Figure 2a** - 2b**). The vacuum infiltration apparatus consists of a vacuum oven which forms the infiltration chamber (Figure 2f). The oven is connected to a pump via a vacuum reservoir. This greatly reduces the time required to achieve the desired vacuum in the infiltration chamber. Plants are secured in a bespoke holder and inverted into a 10 L stainless-steel water bath filled with *A. tumefaciens *suspension (Figure 2a** - 2c**). Complete immersion of the aerial parts of the plants is important for efficient infiltration. The water bath is then placed within the infiltration chamber (Figure 2d** - 2e**), and the vacuum applied to draw the air out of the leaf interstitial spaces. Once the pressure has been reduced by 880 mbar (a process which takes approximately 1 min) the infiltration chamber is brought quickly back to atmospheric pressure over 20 - 30 s by opening the oven inlet valve, whereupon infiltration is complete.

Five days after infiltration the plant material is ready for harvesting and subsequent extraction and isolation of the desired product(s). From this point the process is simply one of natural product extraction and purification from leaf material, a workflow that is familiar to any natural product chemist. Many different methods for initial extraction and subsequent purification exist^15^. The most appropriate choice of methods and the exact conditions used is highly dependent on the particular chemical properties of the compound of interest, in addition to the availability of skills and/or equipment. It is not possible to include in this protocol a fully generalizable, step-by-step method for the downstream processing of harvested plant leaf material to isolated product that could be followed blindly for any triterpene product of interest, nor would it be appropriate to attempt to do so. However, this protocol will provide an overview of the basic workflow used in our laboratory and some methods for the early stages of the process, which in our experience have proved generalizable for most oxygenated aglycone triterpene products. This includes two relatively uncommon techniques, namely, Pressurized Solvent Extraction (PSE), and a convenient heterogenous phase method for chlorophyll removal using a strongly basic ion exchange resin.

PSE is a highly efficient technique for the extraction of small organic molecules from solid matrixes. Extractions are performed under pressure (*ca.* 100 - 200 bar), the main advantage being the ability to use alleviated temperatures which exceed the boiling point of the extraction solvent. This can significantly reduce the time and amount of solvent required to achieve an exhaustive extraction, when compared to other hot solvent techniques such as simple refluxing or Soxhlet extractions^16^. Commercial bench-top PSE instruments are available, which utilize interchangeable extraction cells, and automated solvent handling, heating, and monitoring. This makes this technique extremely convenient. It is also arguably less hazardous, particularly for operators with limited practical chemistry experience.

Saponification of crude leaf extracts under reflux followed by liquid/liquid partitioning is a common technique for the bulk removal of chlorophylls prior to subsequent purification or analysis. However, this can often be operationally demanding on larger scales. Furthermore, detection of the interface, or product loss due to the formation of emulsions can be problematic. The use of strongly basic ion-exchange resins to perform heterogenous phase hydrolysis can serve as a convenient alternative. The pigmented portion of the hydrolyzed chlorophyll remains adhered to the resin and can be simply removed by filtration. This protocol utilizes a preparative scale adaptation of a previously reported analytic procedure^17^ that employs a commercially available basic ion-exchange resin.

Below we describe a detailed and rapid protocol for the preparative scale production of triterpenes utilizing this plant-based platform. This protocol is used routinely in our laboratory to prepare tens to hundreds of milligrams of isolated triterpene product for applications such a structural characterization by nuclear magnetic resonance (NMR) spectroscopy, and/or further study in functional assays.

## Protocol

**Note: **Before following this protocol, become familiar with the material safety data for all substances used, and take appropriate safety precautions. These include but are not limited to: wearing safety glasses when working with glass under vacuum, and handling dry silica gel in a fume cupboard. It is also essential that local legislation concerning working with transgenic material is checked and observed, including following appropriate containment procedures.

### 1. Generate *A. tumefaciens* Strains for Infiltration

**Note: **We provide here a simplified description of the steps required to generate suitable *A. tumefaciens *strains for transient expression. Further details of these steps are described by Sainsbury *et al.*^8^.

1. Amplify by PCR or synthesize the protein coding region of the gene of interest flanked by 5’ and 3’ attB sequences suitable for generation of an entry clone for use with a site-specific recombination cloning protocol^18^,^19^.
  1. Use Forward primer: **GGGGACAAGTTTGTACAAAAAAGCAGGCTTA**NNNNNNNNNNNNNNNNNN, Reverse primer: **GGGGACCACTTTGTACAAGAAAGCTGGGTA**NNNNNNNNNNNNNNNNNN (NNNN… = sequence specific sequence).
  2. Use (representative) PCR conditions: Reaction mixture (25 µL, total), buffer (5x, 5 µL, see **Table of Materials**), dNTPs (10 mM, 0.5 µL), Forward primer (10 µM, 1.25 µL), Reverse primer (10 µM, 1.25 µL), Template DNA (variable concentration, 50 – 250 ng, 0.5 µL), DNA polymerase (2000 U/mL, 0.25 µL, see **Table of Materials**), Nuclease-free water (to 25 µL). Run the thermocycler as follows: initial denaturation (98 °C, 30 s); start cycle (98 °C, 30 s; 45 – 72 °C, 20 s; 72 °C, 30 s/kb) end cycle; 35 cycles; final extension (72 °C, 10 min).
  3. Follow a standard site-specific recombination cloning protocol^18^,^19^ to generate an entry clone using any non-kanamycin-based entry vector (we use pDONR207 which is commercially available). **Note: **The integrity of the entry clone should be confirmed by sequencing, before using to generate the pEAQ-*HT*-DEST1 expression construct. The pEAQ-*HT*-DEST1 is available on request from the Lomonossoff laboratory at the John Innes Centre in Norwich, UK.
  4. Isolate the pEAQ-*HT*-DEST1 vector from the expression clone generated in step 1.1.3, and store at -20 °C prior to use in step 1.3. **Note: **Any commercially available convenient spin column-based plasmid purification kit designed for extracting plasmids from cloning strains of *E. coli* is suitable. Follow the specific instructions of the chosen plasmid purification kit (see **Table of Materials**).
2. Prepare chemically competent *A. tumefaciens* for transformation.
  1. Inoculate a selective LB (Luria-Bertani medium: 10 g/L bacto-tryptone, 10 g/L NaCl, and 5 g/L yeast extract, agar 10 g/L pH 7.0) agar plate (50 μg/mL rifampicin, 100 μg/mL streptomycin), by streaking *A. tumefaciens* LBA4404 from a glycerol stock culture. Incubate overnight at 28 °C in a standing incubator.
  2. Inoculate 50 mL of selective liquid LB media (50 μg/mL rifampicin, 100 μg/mL streptomycin) with a sample of *A. tumefaciens* LBA4404 cells from step 1.2.1. Incubate overnight at 28 °C in a shaking incubator at 200 rpm.
  3. Cool the culture from step 1.2.2 on ice for 10 min and then pellet the *A. tumefaciens* cells by centrifugation at 4 °C and 4500 x g for 5 min (discard the supernatant).
  4. Resuspend the pellet from step 1.2.3 in 1 mL of ice-cold 20 mM aqueous CaCl_2_ solution and repeat the centrifugation step from step 1.2.3 (discard the supernatant).
  5. Resuspend the pellet from step 1.2.4 in 1 mL of ice-cold 20 mM aqueous CaCl_2_ solution and aliquot the resulting suspension in to 50 µL batches. Flash freeze the aliquots in liquid N_2_ and store at -80 °C prior to use.
3. Transform the prepared *A. tumefaciens* LBA4404 with the isolated pEAQ-*HT*-DEST1 vector generated in step 1.2.
  1. Thaw 50 µL of* A. tumefaciens *suspension generated in step 1.2 on ice, and incubate with 100 ‒ 200 ng of isolated pEAQ-*HT*-DEST1 vector, generated in step 1.1, at 0 °C for 5 min.
  2. Cold shock the incubated suspension from step 1.3.1 in liquid N_2_ for 30 s, then allow to warm to room temperature.
  3. Add 200 μL of SOC medium (SOC: 20 g/L bacto-tryptone, 5 g/L yeast extract, 0.58 g/L NaCl, 0.19 g/L KCl, 2.03 g/L MgCl_2_, 2.46 g/L magnesium sulfate 7-hydrate, 3.6 g/L glucose) to the cold shocked suspension from step 1.3.2 and incubate for 4 h at 28 °C in a shaking incubator at 200 rpm.
  4. Inoculate a selective LB agar plate (50 μg/mL rifampicin, 50 μg/mL kanamycin, 100 μg/mL streptomycin) with the SOC culture from step 1.3.3. Spread thoroughly, and incubate for 3 days at 28 °C in a standing incubator.
  5. Inoculate 5 mL of selective LB media (50 μg/mL rifampicin, 50 μg/mL kanamycin, 100 μg/mL streptomycin) with a single colony from step 1.3.4. Incubate overnight at 28 °C in a shaking incubator at 200 rpm.
  6. Add 200 μL of glycerol to 1 mL of the liquid culture from step 1.3.5, mix thoroughly and store at -80 °C. **Note: **This culture can be revived for future experiments by streaking onto LB agar plates with appropriate antibiotics (described below).

### 2. Prepare Infiltration Suspension

1. Inoculate a selective LB agar plate with streaks of the desired *A. tumefaciens* strains from the glycerol stock cultures generated in step 1. Incubate overnight at 28 °C in a standing incubator. **Note:** A strain carrying *tHMGR *within a pEAQ-*HT*-DEST1 vector may also be included.
  1. Individually inoculate 50 mL of selective LB media with a sample of each *A. tumefaciens* strain grown in step 2.1 and incubate overnight at 28 °C in a shaking incubator at 200 rpm.
  2. Individually transfer the 50 mL cultures from step 2.1.1 to 1000 mL of selective LB media (rifampicin 50 μg/mL, kanamycin, 50 μg/mL streptomycin 100 μg/mL) and incubate overnight at 28 °C in a shaking incubator at 200 rpm.
  3. Individually pellet the *A. tumefaciens* by centrifugation (4000 x g), from the liquid cultures generated in step 2.1.2 (discard the supernatant).
  4. Individually resuspend the pellets from step 2.1.3 in 50 mL of freshly prepared MMA buffer (MMA Buffer: 10 mM 2-(N-morpholino)-ethanesulphonic acid, pH 5.6 (KOH), 10 mM MgCl_2_, 150 μM 3’5’-dimethoxy-4’-acetophenone) and incubate at room temperature in the dark for 1 h.
  5. Combine the appropriate volume of each *A. tumefaciens *MMA suspension (generated in step 2.1.4) to produce a final OD_600_ of at least 0.2 for each strain in a 10 L total final volume.
  6. Add additional MMA buffer to the combined suspensions generated in step 2.1.5 to a final volume of 1 L (use immediately in step 3). **Note:** It is advisable to prepare an additional 2 L of infiltration suspension for maintaining the liquid level during the infiltration process.
  7. Prepare 1 L of 10x strength MMA buffer.

### 3. Perform Vacuum Infiltration

**Note:** See Figure 2 for a description of vacuum infiltration apparatus construction. Use 5-week-old *N. benthamiana* plants in 9 cm x 9 cm pots for the proceeding steps. Seedlings should be sown in F1 compost (*e.g.* from Levington) and grown for 2 weeks before being transferred to individual pots containing F2 compost. Plants should be grown in a greenhouse at 25 °C, with 16 h per day of light (Use supplementary lighting when required), water daily, maximum density of 100 plants/m^2^.

1. Transfer the 1 L of infiltration suspension generated in step 2 and the 1 L of 10x strength MMA buffer to the infiltration bath, followed by an additional 8 L of water.
  1. Remove the detachable wings of the bespoke plant holder, insert 4 plants (5-week-old plants see preceding note), and reattach the wings. Invert the holder and place on top of the infiltration bath so as to submerge the leaves in the infiltration suspension. **Note:** Ensure the suspension level reaches the top surface of the plant holder. Raise the level with the addition of extra infiltration suspension if required.
  2. Transfer the infiltration bath with submerged plants to the infiltration chamber and close the door. Ensure the air intake valve is closed on the infiltrator. Turn on the vacuum pump and open the inline valve to the vacuum reservoir followed by vacuum intake valve on the infiltration chamber.
  3. Once the pressure in the infiltration chamber has reduced by 880 mbar, shut the vacuum intake valve, and open the air intake valve. Once the infiltration chamber has returned to atmospheric pressure, open the door, and remove the infiltration bath.
  4. Raise the plant holder till the plants are no longer submerged in the infiltration suspension, then gently shake the holder to allow excess suspension to run off the leaves back into the infiltration bath.
  5. Return the holder to the upright position, remove the detachable wings, and remove the plants.
  6. Repeat steps 3.1.1 to 3.1.5 until the desired number of plants have been infiltrated.
  7. Autoclave the used infiltration suspension prior to disposal.
  8. Autoclave the infiltration bath prior to reuse in subsequent experiments.
  9. Sterilize the interior of the infiltration chamber by cleaning with 70% ethanol and leave to air dry prior to reuse in subsequent experiments.

### 4. Grow the Infiltrated Plants

1. Arrange the infiltrated plants from step 3 at a maximum density of 100 plants/m^2^ in a greenhouse.
2. Grow for 5 days at 25 °C and 16 h per day of light (use supplementary lighting when required), and water daily.

### 5. Harvest the Infiltrated Leaves

1. After 5 days of growth, harvest the leaves.
2. Autoclave waste plant material, pots, and soil prior to disposal.

### 6. Dry the Harvested Leaves

**Note: **The exact lyophilization procedure described below depends on the nature of the available lyophilization apparatus available.

1. Lyophilize the harvested leaves until dry.
  1. Place the harvested leaves in a 2 mm-thick polypropylene bag. Freeze the harvested leaves by storing in a -80 °C freezer for 30 min.
  2. Perforate the bag with many small holes using a pin. Place the bagged frozen leaves in the central chamber of the lyophilizer. Ensure all flask attachment taps are closed and then switch on the lyophilizer. Ensure the condenser reaches at least -50 °C and the pressure reduces to at least 0.15 mbar, and then leave overnight.
  3. Switch off the lyophilizer and vent the vacuum by opening a flask attachment tap. Remove the dried leaves from the central chamber, and proceed to step 7.

### 7. Pressurized Solvent Extraction

**Note:** The proceeding steps refer to use of a commercially available PSE instrument (see **Table of Materials**). Please refer to the manufacturer’s literature for more detailed information on operation. The instrument carries out 4 extractions in parallel.

1. Grind the dried leaves into a course powder via a convenient method (*e.g.,* for most triterpene compounds of interest use a domestic food processor, or manually crush the leaves while contained within a bag). **Note: **Grinding with a pestle and mortar under liquid nitrogen is not usually necessary.
  1. Mix the leaf powder with quartz sand (0.3 ‒ 0.9 mm, 20% by volume) in a suitable container; this acts as a dispersant and improves the efficiency of the extraction process.
2. Prepare 4 extraction cells (120 mL) for filling. For this, invert the extraction cells and insert the glass fiber filter, followed by the metal frit and holding plug. Return the extraction cells to the upright position and add a 1 cm deep layer of quartz sand (0.3 ‒ 0.9 mm).
3. Fill the extraction cells.
  1. Fill the extraction cells with the prepared leaf powder generated in step 7.1 up to the fill line. If needed, gently compress the powder during this process to facilitate addition of a greater amount in each extraction cell.
  2. Add a small layer of quartz sand (0.3 ‒ 0.9 mm) to the top of the packed powder and insert the top cellulose filter.
  3. Insert the packed extraction cells in to the PSE instrument and run the desired method. Use the following settings and material; Solvent: 100% ethyl acetate; temperature: 100 °C, pressure: 130 bar. Run 3 cycles as follows: cycle 1: 0 min hold time, cycle 2 and 3: 5 min hold time, end with a 2 min solvent flush, and 12 min nitrogen gas flush. **Note: **It is advisable to first optimize the extraction method on a small scale. However, the following method is often sufficient for most oxidized triterpene aglycones. The liquor from each extraction cell can be combined in the same external collection vessel by specifying the waste line as the extraction destination, instead of the standard individual collection lines. Be aware the amount of solvent used is dependent on the packing of the extraction cells and the set temperature and pressure. The above method typically generates approximately 800 mL of extraction liquor for 4 parallel extractions.
  4. Repeat steps 7.2 to 7.3.3 until all of the leaf powder has been processed.
  5. Concentrate the combined extraction liquor to dryness, via rotary evaporation under vacuum. Check for the expected output (*i.e.*, dark green slurry). **Note:** The exact procedure may vary depending on the set up of the available rotary evaporator. Always wear eye protection when performing rotary evaporation, and check glassware for damage before subjecting to vacuum conditions.
    1. Turn on the chiller recycler, and allow the heat transfer liquid to cool to at least 5 °C. Turn on the water bath, and set the temperature to 35 °C.
    2. Transfer the extraction liquor to an evaporation flask (do not fill more than half the volume of the flask). Concentrate the liquor batch-wise (in to the same flask) if the total volume exceeds this. Attach the filled evaporation flask to the vapor-duct of the rotary evaporator (secure with a joint clip).
    3. Adjust the flask lift position and angle, so as to submerge the bottom third of the evaporation flask in the water bath at an approximately 45° angle. Start the flask spinning at a speed which begins to spread the extraction liquor up the walls of the flask.
    4. Ensure the vent tap is closed and begin to reduce the pressure (using the controller of the vacuum pump) until a moderate drip is observed between the condenser and receiver flask. **Note:** Do not allow the extraction liquor to start to boil. If this occurs reduce the strength of the vacuum to bring the distillation under control.
    5. Monitor and adjust the vacuum strength and spin speed as appropriate until all the solvent is evaporated.

### 8. Basic Ion Exchange Resin Treatment for Removal of Chlorophylls

**Note: **The quantities quoted in the following steps assume an original working scale of 100 ‒ 150 plants. Step 8 is not suitable for products containing carboxylic acid groups, or functional groups that would be hydrolyzed under basic conditions such as esters.

1. Dissolve the crude extract generated in step 7 in a minimal volume of ethanol.
2. Add 50 mL of basic ion exchange resin beads (see **Table of Materials**) to the output of step 7.1.1, and shake at room temperature for 30 min. **Note: **Do not substitute shaking for the use of stirring apparatus. Magnetic or mechanical overhead stirring can compromise the integrity of the resin beads. Suitable agitation can also be conveniently achieved by slow rotation of reaction flask on a rotary evaporator with the vacuum set at atmospheric pressure or disconnected.
3. Add an additional 50 mL of basic ion exchange resin beads every 30 min until the reaction has gone to completion; the basic ion exchange resin beads will change from pink to green in color, and the liquid phase will change from green to orange or a murky brown color depending on the scale.
  1. If in doubt that the reaction has gone to completion, sample 0.5 mL of the liquid. Filter the sample through a small column of diatomaceous earth and observe the color of the filtrate; the reaction is usually complete within 1.5 h.
4. Filter the reaction mixture through a short column of diatomaceous earth.
  1. Perform this via vacuum filtration, or via a glass flash chromatography column using hand bellows to apply pressure. If filtration rate slows, disturb the top of the diatomaceous earth pad with a stirring rod. Collect the filtrate.
5. Rinse the collected resins beads with ethanol, followed by 1:1 ethanol:hexane, and finally hexane. Use a rinse volume sufficient to resuspend the beads on the top of the diatomaceous earth column.
6. Combine the filtrate from step 8.4, and the rinses from step 8.5, and concentrate to dryness by rotary evaporation under vacuum.

### 9. Initial Rough Fractionation

**Note:** The following method is usually appropriate for typical oxidized triterpene aglycones, and employs an automated flash chromatography instrument. Refer to the manufacturer’s literature for more details on operation.

1. Adsorb the crude product generated in step 8 onto flash grade silica gel (pore size: 60 Å, particle size: 35 ‒ 75 μm) for application to a flash chromatography cartridge via dry loading methodology.
  1. Dissolve the crude product generated in step 8 in a minimal volume of suitable organic solvent. Ensure complete dissolution. **Note: **Dichloromethane with the addition of a few drops of methanol is usually an appropriate choice of solvent.
  2. Unscrew the top of a 100 g prefilled flash chromatography cartridge (see **Table of Materials**) and remove the insert to reveal the dry loading head space. Use the head space to measure the appropriate volume of silica gel for dry loading.
  3. Transfer the measured volume of silica gel for dry loading to the solution of crude product, then remove the solvent by rotary evaporation under vacuum. Note: The silica gel should return to a free-flowing powder. Towards the end of the evaporation process gentle scraping may be required to free the silica gel from the side of the evaporation flask.
  4. Equilibrate the 100 g flash chromatography cartridge at 100 mL/min with at least 5 column volumes of 100% hexane, but ideally until the cartridge content is fully and uniformly wetted.
  5. Transfer the prepared dry loading silica gel generated in step 9.1.3 to the dry loading head space of the equilibrated 100 g flash chromatography cartridge, by pouring. Suspension in hexane and a wide mouthed pipette can be used to aid transfer of any remaining dry loading silica gel.
  6. Wet the transferred dry loading silica gel bed with 100% hexane to remove air from the dry loading headspace.
  7. Run the following chromatography method: Solvent [A]: hexane, Solvent [B]: ethyl acetate, Flow rate: 100 mL/min, Gradient: 0% [B] to 100% [B] over 3000 mL, Collection: Collect All, Fraction size: 250 mL.
  8. Analyze the fractions by thin layer chromatography (TLC)^8^ or gas chromatography-mass spectrometry (GC-MS)^8^, and concentration to dryness the fraction(s) containing the compound of interest via rotary evaporation under vacuum.
2. Perform downstream fine purification until the desired level of purity is reached. **Note: **Downstream fine purification is entirely compound specific and it is not possible to provide standardized steps (see the discussion section).

## Representative Results

Typical isolated yields are dependent on the enzyme/enzyme combination under investigation, the chemical stability of the target compound, and how challenging the purification process was. We have previously reported isolated yields of β-amyrin derivatives from our combinatorial biosynthesis experiments ranging from 0.12 to 3.87 mg per gram of dry *N. benthamiana* leaf powder. These compounds ranged widely in the level of oxidation, and included unnatural combinations of enzymes (Figure 3)^8^. This protocol is used routinely in our laboratory to produce tens or hundreds of milligrams of purified product for structural characterization by NMR spectroscopy and downstream functional assays. We have also demonstrated the preparative utility of this platform by producing 0.8 g of the triterpene scaffold β-amyrin at a purity of greater than 98%. This experiment used 459 plants, and represents an isolated yield of 3.3 mg per gram of dry *N. benthamiana* leaf powder^8^.

**Figure:**
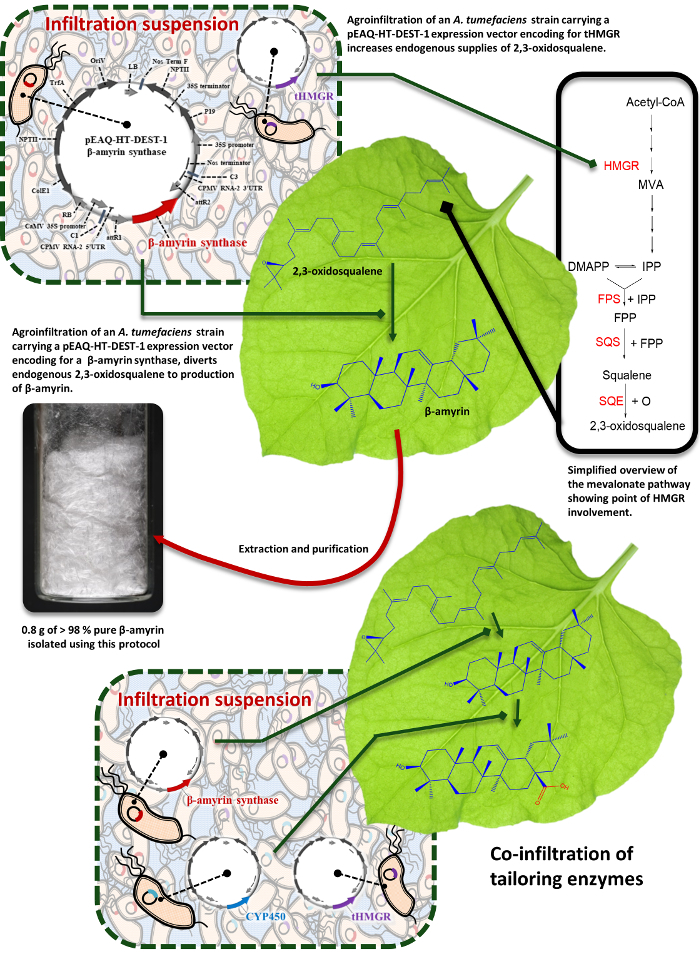

**Figure 1: Schematic summary.** Schematic representation of the process exploiting transient expression of biosynthetic enzymes in *N. benthamiana* leaf to divert endogenous supplies of 2,3-oxidosqualene towards the preparative production of new high-value triterpene products. Please click here to view a larger version of this figure.

**Figure:**
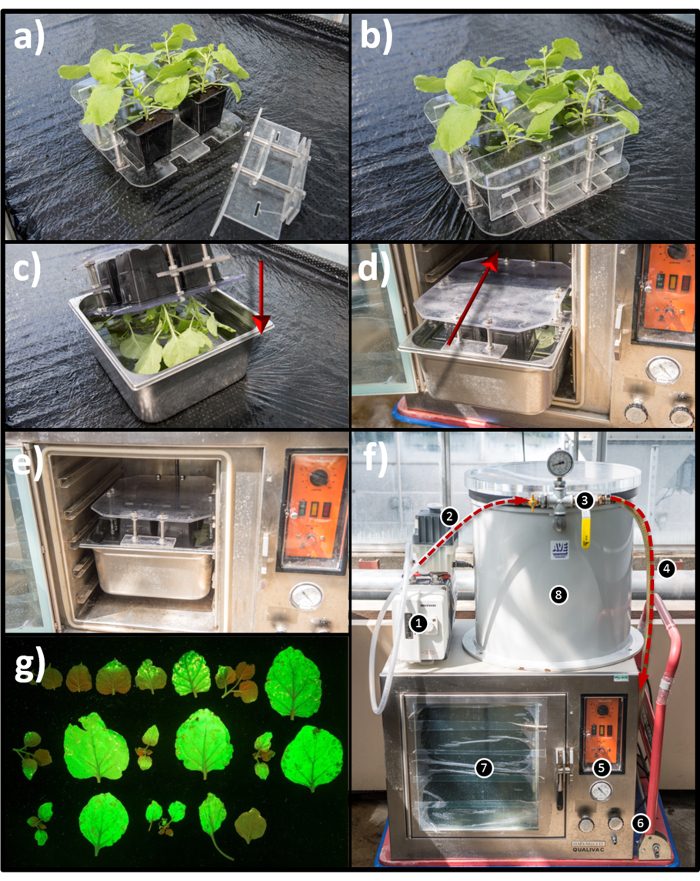

**Figure 2: Vacuum infiltrator construction and process. a)** Bespoke plant holder one wing detached. **b**) Bespoke plant holder wings attached. **c**) Inversion and submersion of plants. **d**) Insertion of the infiltration bath. **e**) Infiltration bath in place within the infiltration chamber. **f**) Complete vacuum infiltrator: (1) Vacuum pump (2) Connection from vacuum pump to vacuum reservoir (3) Vacuum reservoir isolation valve (4) Connection between vacuum reservoir and the infiltration chamber (5) Pressure gauge (6) Air intake valve (7) Infiltration chamber (8) Vacuum reservoir. **g**) Representative leaves from one plant infiltrated with an expression construct for green fluorescent protein (GFP) after five days post-infiltration growth. Please click here to view a larger version of this figure.

**Figure:**
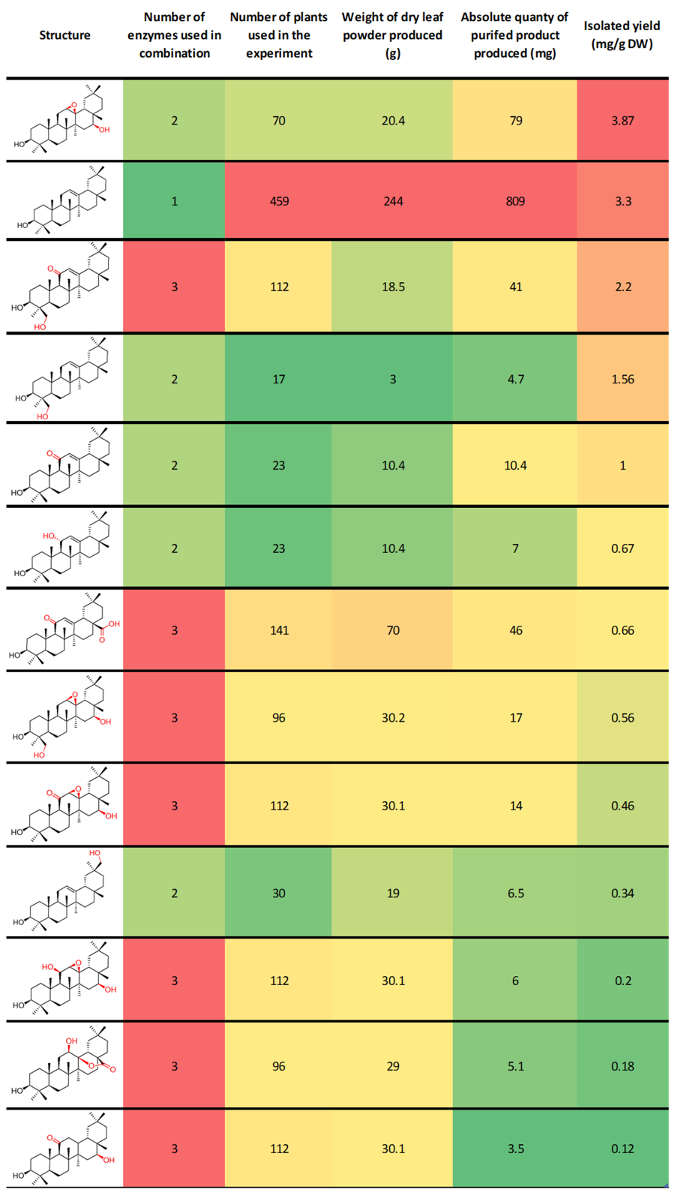

**Figure 3: Previously reported representative results for the preparative production of β-amyrin derivatives using this protocol^8^. **This table is ordered by isolated yields. Columns are conditionally formatted with color indication of relative value. Red = high, Green = low (though intermediate yellow). Please click here to view a larger version of this figure.

## Discussion

High-through-put vacuum infiltration allows this protocol to be used routinely in our laboratory for the preparative production of triterpene compounds of interest for various downstream applications. The vacuum infiltration apparatus described here is easily replicated. Addition of the vacuum reservoir is advisable to decrease the time required to pull the necessary vacuum, however it is possible to simply repurpose an unmodified vacuum oven as the infiltration chamber. Protecting the vacuum pump via addition of a suitable condenser is theoretically sensible, but in our laboratory we have found this to be unnecessary.

Infiltration coverage is compromised if the leaves are not fully immersed in the infiltration suspension. This problem is minimized by ensuring that the level of the suspension reaches the top surface of the plant holder, and that the plant holder is a tight fit for the infiltration bath. Note from Figure 2 that the top surface of the plant holder is recessed in to the infiltration bath when in place. This is achieved by panels on the middle edges of the holder which form the anchor point with the top of the infiltration bath. The infiltration suspension will require periodic additions to maintain the suspension level. This is best achieved by addition of excess infiltration suspension to prevent gradual reduction of the OD_600_ over the course of a large batch-wise infiltration. However, in our hands, substitution for water does not seem to have a noticeable effect on expected yields on a preparative scale, although this has not been quantitively investigated. How many plants can be infiltrated from one batch of infiltration suspension is still an open question. We routinely infiltrate between 100 and 200 plants with a single batch of infiltration suspension. In addition, it is normal for some soil to leach into the infiltration suspension over the course of the infiltration process. This has not been found to have a noticeable effect on infiltration efficacy.

During harvest it is advisable (but not critical) to harvest only the leaves that were infiltrated (some leaves will have grown post-infiltration). Selective harvest prevents dilution with unproductive tissue, which would otherwise increase the ratio of endogenous impurities relative to the compound of interest. This has a nominal impact on the ease of downstream purification, and increases the scale of these processes. When using tHMGR to boost precursor supply, a necrosed phenotype is often observed to develop over the post-infiltration growth period. This is normal and actually aids selective harvest and the downstream drying process. The dried leaf powder can usually be stored in a sealed container in a cool, dry, dark place, but ideally in a desiccator under vacuum. This depends on the stability of the compound of interested. Exercise caution if choosing to store in a -80 °C freezer. Ensure that the dried leaves are in a waterproof container, and on removal from storage, allow this container to warm to room temperature prior to opening. Failure to do so will result in rewetting of the leaves, hindering down-stream processing.

The post-harvest steps in this protocol are provided for illustrative purposes, and to aid those researchers who may have limited practical chemistry experience. They can be substituted for many other natural product extraction/purification techniques. As detailed in the introduction, PSE has many advantages, however the capital cost of commercially available PSE apparatus may be prohibitive, and it is not essential. Basic ion-exchange resin treatment is a very convenient method to replace traditional saponification followed by liquid/liquid partitioning. However, it is not suitable for products containing carboxylic acid groups or functional groups that would be hydrolyzed under basic conditions, such as esters. These products would be expected to be retained on the basic ion exchange resin. However, there is potential to exploit this for a capture and release procedure (not documented here). Where traditional saponification would be appropriate, basic ion-exchange resin treatment serves as a convenient alternative. The chromatography method described in step 9, has been found to be generalizable for the compounds described in Figure 3. However, compounds of increased polarity may require an extended 100% ethyl acetate elution period. With reference to step 9.2, downstream fine purification is entirely compound specific, and is also dependent on scale. Repeated rounds of smaller-scale flash chromatography with optimized gradients, is usually sufficient to achieve a purity appropriate for NMR analysis. On larger scales, crystallization is often a convenient alternative. In difficult cases, preparative or semi-preparative high-performance liquid chromatography (HPLC) can be employed depending on the desired quantity of isolated compound. Examples of downstream purification processes for a range of representative compounds can be found elsewhere^8^.

The current protocol, as presented here, is used routinely in our laboratory, and has proven utility. However, there is still significant scope for further optimization of the expression platform. Work is ongoing in our laboratory to investigate how further pathway engineering and differential control of protein expression levels could improve triterpene production further, while mediating toxicity to host cells. There is also potential to investigate the manipulation of intracellular transport systems^20^,^21^, and the direction of enzymes to different cellular compartments^22^,^23^, avenues which could improve the efficiency of multiple oxidation events. Potential benefits to modifying the underlying morphology of key organelles could also be explored. For example, overexpression of the membrane domain of *Arabidopsis thaliana* HMGR has been observed to induce hypertrophy of the endoplasmic reticulum in plants^24^; a key site for CYP450 activity. This protocol is ideally suited for the production of milligram to gram-scale quantities of triterpene products, and can be employed in a research setting to access compounds for downstream study (*e.g., *the systematic exploration of structure-activity relationships, and preliminary investigations in to the pharmacodynamic and pharmacokinetic properties of lead compounds of clinical interest). However, the platform is linearly and reliably scalable simply by increasing the number of plants used, and the practicality of transient expression via agroinfiltration has been demonstrated on an industrial scale for the production of pharmaceutical proteins^14^, thus there is potential for this platform to be extended for commercial production of high-value triterpenes. Alternatively, it is also possible to envision the production of stable transformants carrying the finalized desire multigene biosynthetic pathway, allowing the mass cultivation and continued 'farming' of transgenic *N. benthamiana* strains producing different compounds of commercial value.

## Disclosures

The authors have nothing to disclose.
